# Costs Associated with Implementation of Computer-Assisted Clinical Decision Support System for Antenatal and Delivery Care: Case Study of Kassena-Nankana District of Northern Ghana

**DOI:** 10.1371/journal.pone.0106416

**Published:** 2014-09-02

**Authors:** Maxwell Ayindenaba Dalaba, Patricia Akweongo, John Williams, Happiness Pius Saronga, Pencho Tonchev, Rainer Sauerborn, Nathan Mensah, Antje Blank, Jens Kaltschmidt, Svetla Loukanova

**Affiliations:** 1 University of Heidelberg, Institute of Public Health, Heidelberg, Germany; 2 Navrongo Health Research Centre, Navrongo, Ghana; 3 University of Ghana, School of Public Health, Accra, Ghana; 4 Muhimbili University of Health and Allied Sciences, Behavioural Sciences Department, School of Public Health and Social Sciences, Dar es Salaam, Tanzania; 5 Medical University, Surgical Clinic, Pleven, Bulgaria; 6 Department of Clinical Pharmacology and Pharmacoepidemiology, Medizinische Klinik (Krehl Klinik), University Hospital of Heidelberg, Heidelberg, Germany; Monash University, Australia

## Abstract

**Objective:**

This study analyzed cost of implementing computer-assisted Clinical Decision Support System (CDSS) in selected health care centres in Ghana.

**Methods:**

A descriptive cross sectional study was conducted in the Kassena-Nankana district (KND). CDSS was deployed in selected health centres in KND as an intervention to manage patients attending antenatal clinics and the labour ward. The CDSS users were mainly nurses who were trained. Activities and associated costs involved in the implementation of CDSS (pre-intervention and intervention) were collected for the period between 2009–2013 from the provider perspective. The ingredients approach was used for the cost analysis. Costs were grouped into personnel, trainings, overheads (recurrent costs) and equipment costs (capital cost). We calculated cost without annualizing capital cost to represent financial cost and cost with annualizing capital costs to represent economic cost.

**Results:**

Twenty-two trained CDSS users (at least 2 users per health centre) participated in the study. Between April 2012 and March 2013, users managed 5,595 antenatal clients and 872 labour clients using the CDSS. We observed a decrease in the proportion of complications during delivery (pre-intervention 10.74% versus post-intervention 9.64%) and a reduction in the number of maternal deaths (pre-intervention 4 deaths versus post-intervention 1 death). The overall financial cost of CDSS implementation was US$23,316, approximately US$1,060 per CDSS user trained. Of the total cost of implementation, 48% (US$11,272) was pre-intervention cost and intervention cost was 52% (US$12,044). Equipment costs accounted for the largest proportion of financial cost: 34% (US$7,917). When economic cost was considered, total cost of implementation was US$17,128–lower than the financial cost by 26.5%.

**Conclusions:**

The study provides useful information in the implementation of CDSS at health facilities to enhance health workers' adherence to practice guidelines and taking accurate decisions to improve maternal health care.

## Introduction

The widely used approach to improve the skills of health workers and the quality of health service provision is the use of clinical decision support systems [1]–[3]. Clinical decision support systems (CDSS) provide clinicians, staff and patients with knowledge and specific information at the point of health services, intelligently filtered and presented at appropriate time, to enhance decision-making process [4]–[6]. CDSS can provide support to health providers on issues such as preventive care, diagnosis, treatment, monitoring and follow ups [5], [6].

In the light of this, most African countries adopted the WHO guidelines on Pregnancy, Childbirth, Postpartum and Newborn Care (PCPNC) [7] to support health workers in clinical decision making for the improvement of quality of maternal and neonatal care (MNC). However, the real implementation of the paper based guideline is a challenge restricting its potential impact [1], [3].

In Kassena-Nankana District of Northern Ghana (KND), the paper based WHO PCPNC guideline is poorly used and followed by health workers [2]. This limits the quality of antenatal and delivery care in the district [8]. In 2012 as an alternative to the paper based WHO PCPNC guideline, Navrongo Health Research Centre introduced a computer-assisted Clinical Decision Support System (CDSS) in the district. This initiative was within the frame of maternal and neonatal care research project-QUALMAT- funded by the 7^th^ Framework programme of the European Union. Computer-assisted CDSS has been successfully implemented in developed countries and applying it in developing countries is promising [9]. Several studies have shown that computer-assisted CDSS helps in the application of knowledge more effectively and as a result improves provider performance and health care quality [4], [10]–[14].

In addition, a computer-assisted CDSS can support clinicians in reducing medical errors and costs [15].With computer-assisted CDSS, patients are likely to be happy that sophisticated technology are being applied to ensure their safety [16].

The QUALMAT computer-assisted CDSS is designed based on locally adapted WHO PCPNC [7]. The computer-assisted CDSS is hoped to be an easy tool to learn, use, and be a source of guidance and information for antenatal care and childbirth at primary health care facilities [6]. It emphasizes prevention and management of specific health problems such as haemorrhage, hypertensive disorders (pre-eclampsia/eclampsia) and obstructed labour, which are the three important causes of maternal mortality in sub-Saharan Africa [8], [17]–[19]. Therefore the introduction of computer-assisted CDSS has the potential to improve quality of MNC and lead to reduction in maternal morbidity and mortality in the district.

Despite the advantages associated with the use of computer-assisted CDSS, the costs of implementing such a system for ANC and delivery services in primary care facilities in developing countries is unknown. This study describes the activities and the cost (financial and economic cost) of a computer-assisted CDSS implementation in the Kassena-Nakana district of northern Ghana to provide estimates that may be useful in guiding other initiatives for computer-assisted CDSS implementation.

## Materials and Methods

### Ethics Statement

The study was approved by the ethics committee of the University of Heidelberg (S-173/2008) and the Institutional Review Board of the Navrongo Health Research Centre in Ghana (NHRCIRB 085).

### Study setting

The study was done in the Kassena-Nankana District of northern Ghana. The district is located in the northeastern corner of the country bordering Burkina Faso, and occupies an area of about 1,675 square kilometres. As in many parts of the savanna zone of Ghana, poverty is endemic in this district. Due to the erratic nature of rainfall and deteriorating soil quality, harvests are often poor and seasonal food shortages are not unusual. The KND has a population of 150,000 with females constituting 53% [20].

The district has a district hospital located in Navrongo that serves as a referral point for the Kassena-Nankana district, the Builsa district and neighboring towns in Burkina Faso. There are six main health centers, and two community clinics jointly run by the Catholic Diocesan Development Office and the District Health Administration that provide services to the communities. There is one private clinic and 27 functional Community-based Health Planning and Services (CHPS) compounds with resident Community Health Officers (CHOs) offering doorstep services [21].

### Study design

A descriptive cross sectional study was conducted in the Kassena-Nankana district. The study captured data from October 2009 to March 2013. Costs were collected from the programme/provider perspective. All information on activities involved in the implementation of the computer-assisted CDSS and associated costs were collected. These activities included training of computer-assisted CDSS users, buying equipment such as laptops and monitoring and supervision of computer-assisted CDSS users. Computer-assisted CDSS software development costs were not collected and therefore not included in the analysis. The study is part of a wider project assessing the quality of maternal and prenatal care (QUALMAT-Quality of prenatal and maternal care: bridging the know-do gap) using a computer-assisted CDSS. The QUALMAT research project started in 2009 as collaboration between six partners- Centre de Recherche en Santé de Nouna (Burkina Faso), Ghent University (Belgium), Heidelberg University (Germany), Karolinska Institute (Sweden), Muhimbili University of Health and Allied Sciences (Tanzania), and Navrongo Health Research Centre (NHRC, Ghana).

### Selected health facilities

The computer-assisted CDSS is being tested in the six health care centres in the Kassena-Nankana district. Each health centre has at least a nurse, a midwife, and a medical assistant and provides outpatient services and normal deliveries. With regards to maternal health care, the Ghana health service policy stipulates that all health facilities are to use the reproductive health guideline “ National Safe Motherhood Service Protocol (2008)” [19] and the WHO guideline ‘Pregnancy, Childbirth, Postpartum and Newborn Care (PCPNC); A Guide for Essential Practice” [7]. Maternal health care is free of charge for all women accessing care in all public health facilities (including the selected health centres). The maternity services that are provided free of charge include antenatal care, childbirth, caesarean section, management of emergency obstetric conditions, and postnatal care [22].

The selected health centres have basic infrastructure such as utilities (electricity and water) equipment and medical supplies [21]. These health centres are within 2 hours drive to the district hospital where patients can be referred to [6].

### Computer-assisted Clinical Decision Support System (CDSS)

Future users (midwives), medical experts and Information Technology (IT) specialists jointly developed the computer-assisted Clinical Decision Support System (CDSS) within the project and the system was subsequently adapted to the country specifics of Tanzania, Burkina Faso and Ghana. The Ghana computer-assisted CDSS is based on Ghana's reproductive health guideline “ National Safe Motherhood Service Protocol (2008)” [19] and the WHO guideline ‘Pregnancy, Childbirth, Postpartum and Newborn Care (PCPNC); A Guide for Essential Practice” [7] and then accustomed to the study health centres [6]. These guidelines have algorithms for decision-making and management of conditions at all levels of care and for all the stages of pregnancy, delivery and postpartum period [2]. The QUALMAT computer-assisted CDSS translates these decision-making trees into computer-based algorithms to be carefully followed by health care providers. The computer-assisted CDSS supports routine antenatal care and care during delivery and up to 24 hours after delivery. It is developed using 3 main principles: (i) Guidance through routine actions in maternal and perinatal care is provided by checklist to safeguard comprehensive history taking; (ii) Integration of clinical data to detect situations of concern by algorithms which then suggest diagnoses or alert the user about dangerous situations that need attention; (iii) Electronic partograph for observation of the progress of delivery up to 24 hours after delivery. Detailed description on the development of the computer-assisted CDSS and how it works are presented elsewhere [6].

Two software release candidates were piloted and feedback incorporated to produce a final version that was then used for implementation. The final version of the computer-assisted CDSS software was implemented and authorized for use in patient care in the six health centres in April 2012.

### Source of data

A form was designed for the project coordinator to document all activities and associated costs (quantities and unit costs) that occurred during the CDSS implementation. In addition, the project had a financial manager who kept records on all expenditure made on the project. Information on the activities and costs were therefore retrieved from both the project coordinator and the financial manager. We collated information on trainings, meetings, management, monitoring and supervision as well as logistics and transportation involved in the computer-assisted CDSS implementation (Table 1). Data on both quantities and unit costs of resources consumed were gathered for the period October 2009 to April 2013.

**Table 1:** Staff, ANC and referrals per health centre.

	12 months before CDSS intervention		12 months CDSS intervention				
Health care centres	ANC consultation	Referrals	Nurses trained on CDSS	ANC consultation	ANC clients managed with CDSS	Referrals	Referrals based on CDSS
Paga	2174	26	4	2726	505	62	8
Chiana	1400	36	4	1718	1082	39	7
Kassena-Nankana East (KNE)	1751	0	4	1492	601	1	1
Sirigu	1493	16	3	1306	708	22	11
Navrongo Health Centre (NHC)	4800	18	3	4933	2312	25	4
Kolgo	725	13	4	679	387	6	9
**Total**	**12343**	**109**	**22**	**12854**	**5595**	**155**	**40**
**Average**	**2057.2**	**18.2**	**3.67**	**2142.3**	**932.50**	**25.8**	**6.67**

For the effects of the CDSS, we collected pre-intervention data to capture 12 months (April 2011 to March 2012) of information on maternal health routine services before computer-assisted CDSS implementation and the post-intervention captured 12 months (April 2012 to March 2013) of data after computer-assisted CDSS implementation. The health centres have books that there record maternal health services provided. We therefore reviewed these books or registers at the health centres and collected data on the number of antenatal consultations, labour cases, deliveries, referrals (due to complications) during ANC visits as well as labour referrals (due to complications). We also collected information on maternal mortality that occurred at the study health centres during the study period.

### Data processing and analysis

The data were entered, cleaned and analyzed using excel. An ingredients approach where quantities of the resources are multiplied by their unit prices was used to calculate costs. Costs were categorized into personnel, trainings, overheads costs (representing recurrent costs) and equipment costs (representing capital cost).

Costs of implementation were further grouped into two phases. The first phase was referred to as the pre-intervention phase. This first phase was defined as all activities and associated costs that occurred before computer-assisted CDSS was commenced for patient care (October 2009 to March 2012 period). The second phase was referred to as the intervention phase and included all the activities and associated costs incurred during the 12 months period of actual use of computer-assisted CDSS for patient care (April 2012 to March 2013). The cost of implementation was calculated by adding the pre-intervention costs and intervention costs.

We calculated cost without annualizing capital cost to represent financial cost of the implementation and cost with annualizing capital costs to allow differential timing of capital costs to represent economic cost. Capital costs (resources with lifespan greater than 1 year) such as equipment (laptops, tables are chairs) were annualized using discount rate of 3% and a useful life of 5 years, consistent with economic evaluation guidelines [23], [24].

Personnel costs were calculated by summing the cost incurred on staff involved in the computer-assisted CDSS intervention during the 12 months period of actual use of computer-assisted CDSS for patient care. The two main staff that were involved were a technical Officer and computer-assisted CDSS users. A technical officer with knowledge in information technology (IT) provided trainings and technical support to the computer-assisted CDSS users. The officer visited the computer-assisted CDSS users fortnightly to monitor and supervise them. The technical officer also downloaded data captured in the computer-assisted CDSS and updated antivirus during the monitoring visits. In addition, whenever a computer-assisted CDSS user moves (transfer, study leave), the officer trains a new user to continue the use of the computer-assisted CDSS. Based on the time spent monitoring, supervising and training the computer-assisted CDSS users (45% of time spent on CDSS activities) and the monthly pay for staff in technical officer category (US$1,000), an agreed amount of US$250 was paid per month to the technical officer. The total cost of the technical officer was therefore calculated by multiplying number of months worked by the monthly allowance.

Not all staff at the health centres were trained to use the CDSS. At least two nurses were trained in each health centre. Each health centre had at least one midwife. All the midwives in the health centres were trained. In addition to the midwives (main CDSS users), some community health nurses (CHN) were trained to support the midwives. These additional nurses were selected in consultation with the in-charges of the study health centres. The basic criteria for selection of these additional nurses for the training were based on their involvement in the provision of antenatal care and delivery in the health centre. Whenever a midwife was transferred or on annual leave or left for further studies, a new person was trained on time for replacement. The staff that were not selected and trained to use the CDSS carried out their normal duties. CDSS users and non-users both abided by the normal work regulations (both start and close work same time). In order not to disrupt the normal duties of the selected CDSS users, training sessions were organized on days and times that were most convenient to the CDSS users.

For the period, 22 computer-assisted CDSS users were trained (16 midwives, and 6 nurses). The government of Ghana pays on average US$626 per month to a midwife, and US$ 404 per month to a community health nurse. We did not include these salary costs in our personnel cost, since this cost was already incurred by the government and the focus was on additional costs. However, in order to motivate the computer-assisted CDSS users to use the system, a token (allowance) of US$31 was given per month to each CDSS user. Cost of computer-assisted CDSS users was therefore calculated by multiplying the number of months worked by the computer-assisted CDSS users by the monthly allowance.

Training costs included all cost incurred during the various training sessions. For the period, a total of six major meetings/training sessions were held. The first meeting was a one day stakeholder meeting, in which the directors of health services, district public health nurses, midwives and other key stakeholders participated. During the meeting, the computer-assisted CDSS concept was discussed and approval and support by the stakeholders were established.

Five training sessions for the computer-assisted CDSS users were held. Each training session lasted two days. The training sessions were facilitated by a technical officer and four support staff (medical officer, midwife and two research officers). The computer-assisted CDSS users were first introduced to basic computer training sessions, as most of them were using computers for the first time. Further specific trainings on computer-assisted CDSS usage (2 trainings: pre-intervention period) and refresher trainings (2 trainings: intervention period) took place. These training workshops aimed to help users understand the algorithm, the algorithm stages and the decision stages of the programme. During the training workshops, demonstrations and hands-on training took place. In addition, each participant was asked to enter an initial and follow- up visit using a simulated patient material. The computer-assisted CDSS users were also taught how to access and use technical support on the software. Participants of these trainings were each paid an allowance of US$11 per training day for their time. This is a standard amount paid by the Ghana Health Service to health workers who participate in trainings of this kind. The cost of training was calculated by summing all the allowances for participants, facilitator and support staff per training session and other expenditure made during the training sessions.

Transportation costs were calculated as hired costs (US$0.45 per km) per vehicle rented for visiting the health centres for monitoring and supervision. The total cost for transportation was therefore calculated by multiplying the kilometre covered by the cost per kilometre.

Equipment costs included all the equipment bought for the computer-assisted CDSS implementation and the associated costs. Six dell laptop computers (specification: 2 GB RAM, 250 GB hard disk drive, Duo core) were purchased and the computer-assisted CDSS software was installed on each of them. These laptops were distributed to each of the six health centres. One additional laptop was bought and the software installed on it, and it served as a backup laptop. In addition, six computer tables and chairs were bought and distributed to the six computer-assisted CDSS health facilities to support computer-assisted CDSS users' work. The total equipment cost was calculated by multiplying the unit cost of items by the quantities. Further analysis was done where laptops, chairs, tables were categorized as capital cost and were annualized using discount rate of 3%, and a useful life of 5 years to determine the economic cost.

Other costs (overheads) including stationary, repairs of computers and other costs incurred in the computer-assisted CDSS implementation were obtained and calculated by multiplying the unit cost of the items by their quantities.

Average cost of training a computer-assisted CDSS user was calculated by dividing the total cost of computer-assisted CDSS implementation by the number of computer-assisted CDSS users trained during the entire study. Computer-assisted CDSS cost per woman managed was calculated by dividing the total cost of implementation by the total number of women managed using computer-assisted CDSS.

One-way sensitivity analysis was conducted to determine whether changes in variables such as discount rate and life expectancy of equipment will change the economic costs of CDSS implementation significantly. Accordingly, we varied the discount rate from 3% to 5% and 10% as well as the life span for equipment from 5 years to 10 years.

Given that the aim of this analysis was to provide the cost of the computer-assisted CDSS implementation, which would be of use to health providers/policy makers considering the use of computer-assisted CDSS within health centres, we did not include health centre specific costs such as actual salaries of computer-assisted CDSS users (midwives and CHNs), capital costs (such as buildings of the health centres) in the analysis as these were already costs incurred by the health centres. Only additional costs not already borne by the health centres were included.

In addition, cost of developing the CDSS software, research cost such as cost of conducting cross sectional surveys and qualitative studies and other costs not related to computer-assisted CDSS implementation were not included.

Costs results are presented by: pre-intervention cost (cost from October 2009 to March 2012); intervention representing the cost incurred over the course of the one year intervention (from April 2012 to March 2013); and cost of implementation representing overall cost (pre-intervention plus intervention cost). Costs were also presented by health centres. The personnel, training, and overhead costs were allocating to the various health centres based on the number of staff trained per health centre. Transportation cost allocation was based on the distance from the research centre to the health centres. All costs were collected in local currency, Ghana cedis (GH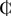) but results are presented in US dollars (US$). We used the average exchange rate for 2012 (1US$ =  GH
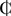
1.8).

## Results

### Computer-assisted CDSS and management of antenatal and delivery patients

Between October 2009 and March 2013, twenty-two nurses were trained (16 midwives, and 6 CHNs) on computer-assisted CDSS to manage antenatal and labour patients. The prime users were the midwives with the CHN providing assistance. Out of 22 nurses, about 80% (18) had no basic computer knowledge before they were introduced to computer-assisted CDSS. However, with the intensive training, all were able to attain the necessary skills to use the computer-assisted CDSS. Twelve months before the CDSS implementation (pre-intervention), the total number of ANC consultations was 12,343 (average: 5,057). At the end of 12 months of CDSS implementation (April 2012 to March 2013), ANC consultations increased to 12, 854 (average: 2,142) (Table 1). Of the total ANC attendance, 0.88% (109) of the women were diagnosed to have pregnancy complications were referred to a hospital for care. At the end of 12 months of CDSS implementation, proportion of complications diagnosed and referred during ANC visits in the health centres (with and without the use of CDSS software) increased to 1.21% (155) (Table 1). The change (absolute difference: 0.33%) in the proportion of complications was statistically significant (pre-intervention 0.88% versus post-intervention 1.21%; P-value = 0.010).

It must be noted that not all clients were managed with the CDSS. There were occasions that the main CDSS user was not available during ANC consultations or/and delivery and for that matter clients were not managed using the CDSS. Out of the total ANC consultation patients, 5,595 (44%) were managed in line with guidelines by a midwife using the computer-assisted CDSS.

During the 12 months pre-intervention period, the number of labour cases registered in the six health centres was 1,769 (average: 295; range: 115–413). Of the total labour cases, 10.74% (190) had complications during labour/delivery and were referred to a hospital for care. At the end of the twelve months CDSS intervention, the proportion of women who had complications during labour or delivery and were referred decreased to 9.6% (141), though the difference was not statistically significant (P-value = 0.287). Comparing labour clients and deliveries, the proportion of deliveries attended by skilled midwives increased from 89% (pre-intervention) to 90% (post-intervention), though not statistically significant (P value = 0.3870). Out of 1,463 labour patients recorded during the intervention period, 872 (60%) were managed using computer-assisted CDSS (Table 2). This represented an adherence to the use of the CDSS to be 44% for ANC and 60% for labour patients. Based on the computer-assisted CDSS recommendation, 40 (0.7%) antenatal clients and 10 (1.2%) labour clients were referred to a hospital for further management. In the six health centres, two maternal deaths were recorded at pre-intervention period and one death was recorded at post-intervention.

**Table 2:** Labour clients, deliveries and referrals per health centre.

	12 months before CDSS intervention			12 months CDSS intervention				
Health care centres	Labour clients	Referrals during labour	Deliveries	Labour clients	Clients managed using CDSS	Referrals during labour/delivery	Deliveries	CDSS ref with CDSS
Paga	383	31	352	338	130	46	292	2
Chiana	336	45	291	265	198	37	228	2
Kassena-Nankan East (KNE)	267	15	252	261	136	15	246	1
Sirigu	413	33	380	331	194	18	313	2
Navrongo Health Centre (NHC)	255	39	216	159	102	8	151	1
Kolgo	115	27	88	109	112	17	92	2
**Total**	**1769**	**190**	**1579**	**1463**	**872**	**141**	**1322**	**10**
**Average**	**294.8**	**31.7**	**263.2**	**243.8**	**145.33**	**23.5**	**220.3**	**1.67**

The average time spent by a midwife per antenatal consultation without the use of CDSS was about 10 minutes. Using the CDSS doubled the average time spent on an ANC client to about 20 minutes. Due to problems related to data collection, we were not able to estimate the time spent by midwives on labour clients before and during CDSS intervention.

### Financial costs of computer-assisted CDSS intervention

Table 3 presents the capital and recurrent financial costs of computer-assisted CDSS implementation. The financial costs aimed to show the money spent on the implementation, for that matter capital costs have not been annualized. The overall financial cost of the computer-assisted CDSS implementation (capital and recurrent costs) from 2009 to 2013 was US$23,316. This is approximately US$1,060 per a nurse (computer-assisted CDSS user) trained. However for correctly following the clinical guidelines to examine an antenatal woman using the computer-assisted CDSS the cost was US$4.17 per woman if all 22 users spent their time on antenatal patients. On the other hand if all 22 computer-assisted CDSS users spent their time in the labour ward the cost was observed to be US$26.74, which is about 6.4 times higher than the cost per antenatal case. Of the total cost of implementation, 48% (US$11,272) was pre-intervention cost while US$12,044, representing 52% of the total implementation cost was the intervention phase costs. Out of the total intervention cost, 55% (US$6,667) was personnel cost.

**Table 3:** Financial costs distribution of computer-assisted CDSS implementation (US$).

Cost category	Sub-category	Items	Pre-intervention cost (US$)	During intervention cost (US$)	Overall intervention cost (US$)	Percentage of total cost (%)
**Recurrent cost**	Personnel	Technical officer, computer-assisted CDSS users	0	6,667	6,667	28.6
	Meeting and Training	Stakeholder meeting, basic computer training, computer-assisted CDSS training, refresher trainings	2,365	468	2,833	12.1
	Transportation	Hiring of vehicles and motorbikes	400	1,580	1,980	8.5
	Others (overheads)	Stationary, communication, computer repairs etc.	1,889	2,031	3,919	16.8
**Capital cost**	Equipment	Laptops, computer desks and chairs	6,619	1,298	7,917	34
**Total cost**			**11,272**	**12,044**	**23,316**	**100**

Of the total financial cost of implementation, equipment cost accounted for the largest proportion of expenditure (34%), followed by personnel (29%), while training and transportation costs accounted for 12% and 8.5% respectively. About 61% (US$4,807) of the equipment cost was spent on the purchase of the computer-assisted CDSS laptops. By disaggregating the costs, recurrent cost (personnel, training, transportation, overhead costs) was US$15,399 representing 66% of total cost.

### Economic cost of computer-assisted CDSS intervention

The distribution of economic cost is presented in Table 4. Economic costs were based on the financial costs but capital costs were converted to an annual equivalent (annualization) using a useful life of five years and a 3% discount rate. The overall economic cost was US$17,128 representing 26.54% reduction in total cost. Capital cost after annualization decreased to US$1,729, representing 4.6 times reduction in capital cost. Of the total economic cost of implementation, 35% (US$6,099) was pre-intervention cost, while 65% (US$11,029) was the intervention phase costs. Personnel cost accounted for the highest proportion of the total economic cost (39%) and equipment cost accounted for 10% of the total economic cost. The Kolgo health centre had the highest cost of US$3,246.82, due to higher transportation cost (Table 5). The Kolgo health centre is far away from the research centre (about 20 km) compared to the others.

**Table 4:** Economic cost of computer-assisted CDSS Implementation (US$).

Cost category	Sub-category	Items	Pre-intervention cost (US$)	During intervention cost (US$)	Overall intervention cost (US$)	Percentage of total cost (%)
**Recurrent cost**	Personnel	Technical officer, computer-assisted CDSS users	0	6,667	6,667	38.9
	Meeting and Training	Stakeholder meeting, basic computer training, computer-assisted CDSS training, refresher trainings	2,365	468	2,833	16.5
	Transportation	Hiring of vehicles and motorbikes	400	1,580	1,980	11.6
	Others (overheads)	Stationary, communication, computer repairs etc.	1,889	2,031	3,919	22.9
**Capital cost**	Equipment	Laptops, computer desks and chairs	1,445	284	1,729	10.1
**Total**			**6,099**	**11,029**	**17,128**	**100**

**Table 5:** Cost distribution by health centres (Economic costs).

Health care centres	Paga (US$)	Chiana (US$)	Kassena-Nankana East (US$)	Sirigu (US$)	Navrongo Health Centre (US$)	Kolgo (US$)	Total (US$)
Personnel	1212.18	1212.18	1212.18	909.14	909.14	1212.18	6667
Meeting and Training	515.09	515.09	515.09	386.32	386.32	515.09	2833
Transportation	216	390	388	432	36	519	1981
Others (overheads)	712.55	712.55	712.55	534.41	534.41	712.55	3919
Equipment	288	288	288	288	288	288	1728
**Total**	**2943.82**	**3117.82**	**3115.82**	**2549.86**	**2153.86**	**3246.82**	**17128**
**average**	**588.76**	**623.56**	**623.16**	**509.97**	**430.77**	**649.36**	**3425.60**

Results from sensitivity analysis (Table 6) showed that changing the discount rate applied to equipment from 3% to 5% and 10% has little impact on costs, as total cost, cost per ANC woman and cost per labour woman increased marginally (less than 3%). Likewise, increasing the life expectancy of equipment from 5 years to 10 years increased costs by less than 5%.

**Table 6:** Sensitivity analysis.

Variables	Total cost	Cost per ANC provided	Cost per labour care provided	Cost per nurse trained
**Discount rates**				
No discount rate (financial cost)	23,316	4.17	26.74	1059.82
Using 3% discount rate and 5 years life span (Economic cost)	17,128	3.06	19.64	778.55
Percentage change (Financial vs. Economic cost)	26.54	26.62	26.55	26.54
Using 5% discount rate and 5 years life span	17,228	3.08	19.76	783.1
Percentage change (3% vs. 5% discount rate)	0.59%	0.65%	0.59%	0.58%
Using 10% discount rate and 5 years life span	17,488	3.13	20.06	794.92
Percentage change(3% vs. 10% discount rate)	2.10%	2.29%	2.09%	2.10%
**Change in life span**				
Using 10 years life span and 3% discount rate	16,328	2.92	18.72	742.17
Percentage change(5 vs. 10 years life span)	4.67%	4.58%	4.69%	4.67%

## Discussion

This study analyzed cost for implementation of computer-assisted Clinical Decision Support System (CDSS) in six primary health centres in the Kassena-Nankana district of northern Ghana. The findings indicated that the total financial cost of implementing a computer-assisted CDSS was US$23,316 while the average financial cost per antenatal case using computer-assisted CDSS was $4.17 as well as the per labour case was $26.7. About US$1,060 was needed to train a nurse to use a computer-assisted CDSS. However, due to economies of scale the unit costs are likely to fall over time.

In this study, it is important to note that the greatest proportion of the cost of the computer-assisted CDSS implementation was intervention phase costs. This is in the right direction as compared to other projects implementing new technologies such as computer-assisted CDSS, where most of the total costs were spent in the pre-intervention phase [16]. In our study, most of intervention costs resulted from personnel cost (55%). The study was conducted by a research centre (Navrongo Health Research Centre) and therefore it was necessary to provide allowances to the nurses in order to motivate them to participate in the study. Also it was crucial to hire an IT officer to train and give technical support regularly to CDSS users. However, in future when computer-assisted CDSS is well integrated into the routine maternal care, allowances will not be necessary since it would be part of their normal work.

Equipment such as durable laptop computers are the core of the computer-assisted CDSS. We used laptop computers against desk tops because of unstable power supplies (frequent light offs) faced in the study district. Given that large data captured by computers slows down the system, it is desirable to use a computer with a reasonable space (hard drive) and memory (RAM). In our study, equipment costs accounted for the largest proportion of financial cost (34%). This is contrary to other studies in related areas, that rather found cost of personnel accounting for the entire cost of implementing a computer-assisted CDSS [13]. However when economic costs was estimated by spreading the equipment costs over their useful life years, the cost reduced by 26.54%. Personnel cost then accounted for the largest proportion of economic cost (39%). Also the reason why personnel cost accounted for the largest proportion of financial cost in Field's study [13] was that, they included personnel cost of developing the computer-assisted CDSS software in their analysis.

CDSS users were cautioned to take proper care of the laptops to avoid lost or theft. For study period, no computer was stolen. This corroborates with a similar study conducted in Ghana (MOTECH) in which mobile phones were provided to nurses to provide maternal health care and there was no reported loss of a mobile phone [25]. This suggests that there is low risk of equipment such as electronic devices (computers and mobile phones) provided to health workers stolen in Ghana.

Training on the use of the computer-assisted CDSS was an essential component of the computer-assisted CDSS implementation. Though most of the computer-assisted CDSS users (midwives and CHNs) had no/limited computer knowledge, the study demonstrated that with substantial training and regular support, health workers in rural settings can acquire computer skills and apply it on computer-assisted CDSS for antenatal and delivery care. The need for substantial training of computer-assisted CDSS users has also been mentioned in similar studies [13], [26]. Considering the limited computer knowledge among the CDSS users before the intervention, adherence to the use of the system for patient care (44–60%) is encouraging and shows acceptance. A previous study in Burkina Faso reported positive attitudes and willingness of health workers to use modern technologies such as computer-assisted CDSS [27].

In general, there is low computer knowledge among health workers in Ghana which limits computer usage in the various health facilities. However, in recent times, Information and Communications Technology (ICT) teachings in various schools, including health training schools in Ghana have been intensified. Also the Ghana Ministry of health has instituted strategies to improve ICT in the health facilities [28]. Therefore, an improvement in knowledge and attitudes by health workers towards computer related activities such as computer-assisted CDSS and the sustainability of computer approaches in the near future is envisaged.

Computer-assisted CDSS allows nurses to follow thoroughly the algorithm and patient care guidelines and it provides checks which prompt nurses to complete the whole cycle of care at lower health facilities such as health centres. In our study, on average, one midwife was able to provide all the vital care on 44% of ANC clients and 60% of labour clients with the aid of computer-assisted CDSS. The findings of the study showed increase in the proportion of identified pregnancy complications during antenatal consultations. Considering that most maternal deaths are due to delays in recognizing early complications (danger signs) and immediate referrals [7], [19], [29]–[33], this study demonstrates encouraging effect of CDSS in improving quality of care. This corroborates with a study that demonstrated the elimination of delays faced by community midwives/lady health visitors in providing standard healthcare services(pre-post natal) to mother and child in remote areas in Pakistan due to mobile CDSS [34].The CDSS intervention reduced complications during delivery and as a result reduced maternal deaths. Given that maternal mortality of a single mother reduced per capita GDP by US$0.36 per year in Africa [35], it is worth spending on interventions such as computer-assisted CDSS as it has the propensity of improving the management of pregnancy and delivery and as a result reduce the costs associated with managing these consequences.

Computer based is appropriate over the paper-based CDSS because with computer based, patient characteristics and clinical data are matched to a computerized clinical knowledge base and recommendations are then presented to the health provider to make a decision at the point of care [27]. Mobile phone-based CDSS is another form of electronic-based CDSS and an alternative to the paper-based. However, computer-based is preferred over mobile phone-based because of reported problems of small screen size that affects speed of work [36].

Notwithstanding the numerous benefits of CDSS, there are few reported rejections. For instance, a study reported rejection of CDSS by nurses and physicians based primarily on ethical viewpoints [37]. This was a CDSS for decision making for withdrawal of active medical intervention or end-of-life decision making. Another reason for rejection of CDSS by physicians was that it was too simplistic for routine use [38].The QUALMAT CDSS however is not an end-of-life decision making tool. It only alerts, recommends and provides support in decision making to health workers at the primary health care level where there are no doctors. Most of the concerns reported in previous studies by health workers on CDSS were workload related, especially during the initial stages of use of the system [27], [39]. Though in our study, using CDSS increased the time spent to manage patients during ANC, overtime, when users become familiar with the system the time spent is likely to reduce. Another reason why CDSS usage increased time spent on patient was due to double documentation as they had to complete the usual paper based forms in addition to the CDSS. When the system is integrated in routine activities, there would be no/less double documentation. It has been reported in previous studies that the introduction of a computer-based patient record system shortened the time spent by clinicians on patients and simplified reports preparation [40], [41].

Considering the activities involved and the cost distribution in the implementation of the computer-assisted CDSS, adopting the system and integrating it into the routine maternal health care delivery is feasible and it would require marginal additional costs. For instance, given that personnel costs accounted for a high proportion of total costs, if the system is integrated into the normal health care delivery, this cost component would be reduced or eliminated. Also, over time, transportation costs relating to frequent monitoring and supervision of computer-assisted CDSS users will reduce when users became adequately versed with the system. The additional costs that would therefore be required would be computer costs which would be relatively marginal especially considering economic costs.

### Limitations

The costs presented are based on only one- year usage of the computer-assisted CDSS. However, there is the likelihood of additional costs for maintaining the system over time (computer breakdowns, upgrades of software etc.). Staff attrition is a possible additional cost. For instance, when trained computer-assisted CDSS users are transferred or leave for further studies, new users would have to be trained and supported. There is the possibility of declining willingness to use the system over time which can negatively affect the sustainability of the system. However, if the system is well integrated into routine maternal health care activities at the health centres, then it would be mandatory for the system to be used.

This study provided a lower-bound estimate of the total cost of computer-assisted CDSS implementation as we did not include some costs components such as building costs and salaries of the CDSS users. When these costs are included, the total cost will increase substantially. For instance, given that the government pays on average US$626 per month to a midwife, if this cost is included, then the cost of salaries of midwives for the one year period will be US$45,072. When this is added to the total cost of CDSS implementation (US$17,128), the total cost will quadruple (US$62, 200). Nevertheless, the study was based on the assumption that if CDSS is incorporated into routine maternal health care, these costs components that are already catered for by the government will not be additional costs to the health centres.

There is no published study on cost of computer-assisted CDSS implementation in a developing country to compare our results. The few available studies on cost of computer-assisted CDSS were conducted in developed countries [13], [42]. The setting and the study design does not allow meaningful comparison of findings. Also the studies were not related to maternal health care.

## Conclusion

Though maternal mortality is preventable, these deaths continue to drain a nation of its human resources and Gross Domestic Product (GDP). This study revealed that using computer-assisted CDSS to manage antenatal and delivery clients by midwives in a rural setting is possible. The system can reduce complications during delivery and as a result can reduce maternal deaths. A financial cost of approximately US$1,060 is needed to train a nurse to use computer-assisted CDSS for a one year period. The study provides useful cost information in the implementation of computer-assisted CDSS to enhance health workers adherence to practice guidelines and taking accurate decisions to improve maternal and child health quality.
